# A convenient, rapid and efficient method for establishing transgenic lines of *Brassica napus*

**DOI:** 10.1186/s13007-020-00585-6

**Published:** 2020-03-30

**Authors:** Kai Zhang, Jianjie He, Lu Liu, Runda Xie, Lu Qiu, Xicheng Li, Wenjue Yuan, Kang Chen, Yongtai Yin, May Me Me Kyaw, Aye Aye San, Shisheng Li, Xianying Tang, Chunhua Fu, Maoteng Li

**Affiliations:** 1 Department of Biotechnology, College of Life Science and Technology, Huazhong University of Science and Technology, Wuhan, 430074 China; 2 Hubei Collaborative Innovation Center for the Characteristic Resources Exploitation of Dabie Mountains, Huanggang Normal University, Huanggang, 438000 China; 3 College of Life Sciences, South-Central University for Nationalities, Wuhan, 430074 China

**Keywords:** *DsRed*, *Agrobacterium*-mediated hypocotyl transformation, Visual screening, *Brassica napus*

## Abstract

**Background:**

*Brassica napus* is an important oilseed crop that offers a considerable amount of biomass for global vegetable oil production. The establishment of an efficient genetic transformation system with a convenient transgenic-positive screening method is of great importance for gene functional analysis and molecular breeding. However, to our knowledge, there are few of the aforementioned systems available for efficient application in *B. napus*.

**Results:**

Based on the well-established genetic transformation system in *B. napus*, five vectors carrying the red fluorescence protein encoding gene from *Discosoma* sp*.* (*DsRed*) were constructed and integrated into rapeseed via *Agrobacterium*-mediated hypocotyl transformation. An average of 59.1% tissues were marked with red fluorescence by the visual screening method in tissue culture medium, 96.1% of which, on average, were amplified with the objective genes from eight different rapeseed varieties. In addition, the final transgenic-positive efficiency of the rooted plantlets reached up to 90.7% from red fluorescence marked tissues, which was much higher than that in previous reports. Additionally, visual screening could be applicable to seedlings via integration of *DsRed*, including seed coats, roots, hypocotyls and cotyledons during seed germination. These results indicate that the highly efficient genetic transformation system combined with the transgenic-positive visual screening method helps to conveniently and efficiently obtain transgenic-positive rapeseed plantlets.

**Conclusion:**

A rapid, convenient and highly efficient method was developed to obtain transgenic plants, which can help to obtain the largest proportion of transgene-positive regenerated plantlets, thereby avoiding a long period of plant regeneration. The results of this study will benefit gene functional studies especially in high-throughput molecular biology research.

## Background

*Brassica napus* is an important oilseed crop, ranking as the second most important crop for oilseed production worldwide [1], and it was derived from the hybridization between *B. rapa* and *B. oleracea* [2]. To satisfy the increasing demand for oil, it is essential to improve various important economically and agriculturally traits via genetic engineering techniques, which are powerful tools for gene functional analysis and crop improvement [3]. Genetic transformation techniques have promoted the improvement of crop varieties by integration of novel genes to satisfy the needs for high-yield and good-quality crops, including characters for effective oil production [4], herbicide and disease-resistance [5, 6]. It has been successfully used to improve some major crops, such as soybean, maize and cotton [7–9]. New varieties of these aforementioned plants modified by transgenic methods are now planted widely in many countries, introducing great benefits for farmers [10]. However, to our knowledge, the genetic transformation technique in rapeseed is still relatively lower in transformation efficiency and inefficient in positive screening compared with other crops. Therefore, a suitable and favourable genetic transformation system accompanied by a highly efficient screening method is essential for rapeseed breeding and improvement.

To date, several genetic transformation methods have been reported and routinely applied in model plants and major crops, such as *Arabidopsis thaliana*, *Nicotiana benthamiana*, rice, wheat and maize [11–15]. In *B. napus*, various technologies, including PEG-mediated DNA uptake [16, 17], electroporation [18], particle bombardment [19], *Agrobacterium*-mediated transformation and microspore transfection [20, 21], have been used to obtain genetically modified plants. Among these technologies, *Agrobacterium*-mediated transformation is the most general, reliable and effective method [22–24]. Maheshwari et al*.* (2011) investigated the effect of hormonal combinations, donor plant age and explant types on the transgenic frequency and regeneration capacity of plants in four different rapeseed lines (nvigor 5020, Westar, Topas and its microspore derivative-Line 4079) [25]. They found that the transformation frequency was 54.2 and 53.7% in cultivars of Invigor 5020 and Westar, but 16.0 and 13.4% for Topas and Line 4079, respectively. However, due to the diverse genetic transformation ability caused by distinct genetic backgrounds within the cultivars [5], recalcitrance continued to persist in several cultivars which were not capable of being genetically transformed, especially in commercial varieties.

Obtaining highly efficient transgenic-positive regenerated plantlets is mainly dependent on the subsequent selection. Plant regeneration, however, is a time-consuming process that requires a long growth cycle; simultaneously, it often also results in undesired abortions and reduced fertility. Numerous methods have been frequently used for transgenic-positive plant screening, such as antibiotics, PCR, Southern blot analysis, GFP or GUS staining [14, 26]. These methods, however, are tedious, and they are usually performed with leaves, roots or siliques from the regenerated plantlets. Therefore, it is necessary to develop a convenient and efficient screening method to simplify and promote the process of plant transformation. Recently, red fluorescence protein from *Discosoma *sp. (*DsRed*), similar to *Aequorea victoria* green fluorescent protein (GFP) in the secondary structure, has been applied to identify the transgenic seeds by visual screening in plants [15, 27, 28]. Both the excitation and emission wavelength of *DsRed* (554 nm and 586 nm) are longer than that of GFP (480 nm and 505 nm), which could enable the transgenic-positive screening to be more sensitive [29, 30]. Although both *DsRed* and GFP do not affect the vegetative and reproductive growth of plants, GFP is more easily influenced by the intrinsic chlorophyll in plants [31, 32]. Stuitje et al*.* (2003) found that the sensitivity of *DsRed* was higher than that of GFP in *Arabidopsis* [33]. Thus, *DsRed* has been widely used to mark the transgenic pollen, leaf or seed, and even extraplastidic membranes of chloroplasts for visual selection or offspring separation [33–36]. Eckert et al*.* (2005) were the first to implement the expression of *DsRed* and another common reporter protein-GFP in *Leptosphaeria *spp. and *Oculimacula *spp. to observe the interactions between fungal pathogen species and rapeseed in vitro and *in planta* [37]. Zheng et al*.* (2019) investigated the potential of *DsRed*-labelled *Verticillium longisporum* dissemination by seeds of *B. napus* under greenhouse condition, and confirmed the systemic growth of the pathogen from roots to seeds [38]. However, until now, *DsRed* has not been widely used in tissue culture or in the process of seed development.

The objective of the present study was to construct a convenient and highly efficient method to obtain transgenic-positive lines, by combining a rapid transgenic-positive screening technology with a highly efficient *Agrobacterium*-mediated hypocotyl genetic transformation system in *B. napus*. To achieve this goal, highly efficient genetic transformation was conducted in eight rapeseed varieties and five expression vectors. In addition, *DsRed* was introduced to serve as a visual screening marker for screening transgenic-positive plants during tissue culture and seed development. The present study provides a good foundation for future studies focused on the efficient acquisition of high-throughput transgenic-positive lines.

## Materials and methods

### Plant materials and growth conditions

The allotetraploid rapeseed genotypes used included the spring varieties Jia 9709, semi-winter varieties Jia 2016, and six commercial winter varieties including Zhong Shuang 8, Zhong Shuang 11, Zhong You 821, 7633, B 351 and Shan 3B. The seeds of Jia 2016 and Jia 9709 were kindly provided by Prof. Chunyu Zhang from Huazhong Agriculture University (Wuhan, China). The seeds of Zhong Shuang 8, Zhong Shuang 11 and Zhong You 821 were developed and provided by the Institute of oil crops, Chinese Academy of Agricultural Sciences. The seeds of 7633, B 351 and Shan 3B were kindly provided by Prof. Dianrong Li from the Hybrid Rapeseed Center of Shaanxi Province. These rapeseed varieties were cultured in pots (6 × 6 × 9 cm) in a tissue culture room, and all the rooted plantlets were grown in pots (12 cm × 15 cm) in a greenhouse with 2000–2500 lux with 16 h of light and 8 h of dark at 25 ℃. During growth, water was supplied three times a week. Aphids were controlled with imidacloprid (Jiangsu Changqing Biotechnology Co., China) and sticky coloured cards (Chunhe, China).

### Vector construction

For the *Bna*A07g17400D and *Bna*C05g34170D over-expression vectors, the CDS fragments were amplified and then purified with a QIA quick extraction kit (QIAGEN, America) and then linked to pCMABIA-1303 plasmid which was linearized by EcoR I digestion using in-fusion enzyme, as referenced in the In-Fusion® HD Cloning Kit User Manual. For the *Bna*A07g17400D knock-down vector, two regions (i.e., 364 nt-839 nt and 575 nt-986 nt) were designed. And for the *Bna*C05g34170D knock down vector, the region from 220 to 691 nt was designed. The sense and antisense strands of the three fragments were amplified with R1-S-F/R, R1-A-F/R, R2-S-F/R, R2-A-F/R, R3-S-F/R and R3-A-F/R (Additional file 4), respectively. After amplification and purification, they were cloned into p35S-1390 which was linearized with Sac I and SnaB I for the sense and antisense strand construction, respectively. Afterwards, the marker gene *DsRed*, amplified with the designed primers *DsRed*-F/R (Additional file 4), was purified and then cloned into these reconstructed vectors, digested with Hind III. These reconstructed vectors were transformed into *Agrobacterium* strain GV3101, and single positive clones were identified by PCR amplification and then stored at − 80 °C.

## *Agrobacterium tumefaciens*-mediated transformation of hypocotyls in rapeseed

*A.grobacterium tumefaciens*-mediated genetic transformation was performed according to Zhou et al. (2002) with minor modifications [39]. A brief introduction is provided as follows.

First, seed germination. Clean the seed coat with sterilized water for approximately three times, and sterilized with 70% ethanol for 1 min, followed by 50% of 84 disinfectant (the active ingredient is approximately 2.5% NaClO in the ratio of mass-to-volume) for 3 min, and then rinsed 3–5 times with sterilized water. Finally, the seeds were transferred to 1/2 MS medium and germinated for approximately 7 days in a complete dark condition. Second, *Agrobacterium* preparation. Revive GV3101 harbouring the expression vector that stored at − 80 ℃ for approximately 30 min (28 ℃, 180 rpm) at the sixth day after the first step. Then, 50 μl of the revived *Agrobacterium* was inoculated into 20 ml LB liquid medium and cultured at 28 ℃ (180 rpm) for approximately 12 h. Generally, the OD_600_ reached approximately 0.4, and then 2 ml was obtained for centrifugation (3 min, 6000 rpm) to collect the pellet. The supernatant was removed and the pellet rinsed twice with liquid infection medium (LIM, 4.4 g/L MS, 30 g/L sucrose, 200 mM acetosyringone). The resuspended pellet was kept at 4 ℃ for further infection. Third, *Agrobacterium* infection and cocultivation. Hypocotyls were cut to approximately 1 cm into a sterilized plate containing 18 ml pre-added LIM, and then the aforementioned 2 ml of the resuspended pellet was added to the plate and allowed to infect for approximately 10 min. Next, place the infected hypocotyls in the cocultivation medium (4.4 g/L MS, 30 g/L sucrose, 18 g/L mannitol, 1 mg/L 2,4-D, 0.3 mg/L kinetin, 200 mM acetosyringone and 8 g/L agar) for two days in the dark, at 25 °C. Fourth, calli induction. Transfer all the hypocotyls to calli-inducing medium (CIM, 4.4 g/L MS, 30 g/L sucrose, 18 g/L mannitol, 1 mg/L 2,4-D, 0.3 mg/L kinetin, STS (0.1 M Na_2_S_2_O_3_: 0.1 M AgNO_3_ = 4:1), 300 mg/L timentin, 25 mg/L hygromycin and 8 g/L agar) for 20 days at 2000–2500 lux. Fifth, shoots induction. Transfer all the hypocotyls with embryogenic calli into a shoot-inducing medium (SIM, 4.4 g/L MS, 10 g/L glucose, 0.25 g/L xylose, 0.6 mg/L MES, 2.0 mg/L zeatin, 0.1 mg/L IAA, 3 mg/L AgNO_3_, 300 mg/L timentin, 25 mg/L hygromycin and 8 g/L agar) for shoots regeneration, and renewed the medium every 2–3 weeks until at least 3 leaves appeared. Sixth, roots induction and plantlet regeneration. Transfer the induced shoots into root-inducing medium (RIM, 4.4 g/L MS, 10 g/L sucrose, 1 mg/L IBA, 300 mg/L timentin and 8 g/L agar). Then, transplant the roots plantlets with 4–6 leaves into pots with nutritive soil and cultivated in a greenhouse. The semi-winter and winter plantlets should be vernalized in a cooler at 4 °C for approximately 4 weeks before transplanting.

### Fluorescence observation and imaging

The red fluorescence in the calli and shoots on the medium during the preliminary stage were observed with a hand-held green fluorescent flashlight through a red filter in the dark, and the images were then captured with a D7100 camera (Nikon, Japan). The red fluorescence in roots, seed coats, hypocotyls, and cotyledons of seedlings were observed using a laser confocal fluorescence microscope FV1000 (Olympus, Japan). The images were processed and arranged with Photoshop software.

### RNA extraction and real-time qPCR

To analyse the relative expression level of the *DsRed* and *Bna*A07g17400D gene, a set of roots, seed coats, hypocotyls, cotyledons and whole seedlings were collected one week after sowing the seeds on MS medium. Total RNA was extracted using the RNAprep Pure Plant Kit (polysaccharides & polyphenolics-rich) (Tiangen, China), according to the manufacturer’s protocol. Then, approximately 1 µL RNA samples was used to measure the RNA concentration with a NanoDrop 2000 spectrophotometer (Thermo Scientific, USA). The cDNA was synthesized with 1 μg RNA using a ReverTra Ace qPCR RT Master Mix kit with gDNA Remover (Toyobo, Japan) according to the manufacturer’s protocol, and it was stored at − 20 ℃.

The real-time PCR in a total volume of 20 µL were performed with SYBR Green Real-time PCR Master mix (Toyobo, Japan) on a Stepone Plus (ABI, America) according to the provided protocol. Three technical replicates were analysed. The relative expression of each gene was calculated using the ΔΔCt method. The primers used in RT-PCR are listed in Additional file 4.

### DNA extraction, PCR-based identification and Southern blotting in transgenic rapeseed plants

For DNA extraction, calli were collected from the third renewed SIM, and young leaves were collected from greenhouse-grown T1 plantlets and from the field for T3 plants to the seedling stage, respectively. They were frozen in liquid nitrogen and stored in−80 °C. Genomic DNA was extracted with a NuClean PlantGen DNA kit (CWBIO, China) and kept at − 20 ℃.

To check the positive transgenic plants, presence of the *Bna*A07g17400D and *Bna*C05g34170D gene was performed by PCR amplification using the primer pair *Bna*A07g17400D-OE-F/R and *Bna*C05g34170D-OE-F/R for the E1 and E2 transgenic lines, respectively, while the presence of the *DsRed* gene was detected using the primer pair Red-F/R in the three RNAi lines. The primers are listed in Additional file 4.

Southern blotting analysis was performed in transgenic rapeseed plants according to the published methods with a minor modification [40]. Briefly, total genomic DNA was extracted using the standard CTAB method to obtain a large amount of DNA. Then, 30 μg DNA from each sample was digested with EcoR I, which flanked the *DsRed* gene in the E1, E2 and RNAi vector. The digested DNA samples were fractionated on a 0.8% agarose gel and transferred onto a nylon Hybond-N + membrane with a membrane transfer instrument. The *DsRed* PCR product was labelled with digoxigenin and used as a probe for hybridization with the digested DNA on the membrane. The hybridization and detection steps were performed according to the instructions supplied the DIG High Prime DNA Labelling and Detection Starter Kit II.

## Results

### High efficiency of *Agrobacterium*-mediated hypocotyl transformation in rapeseed

**Fig. 1:**
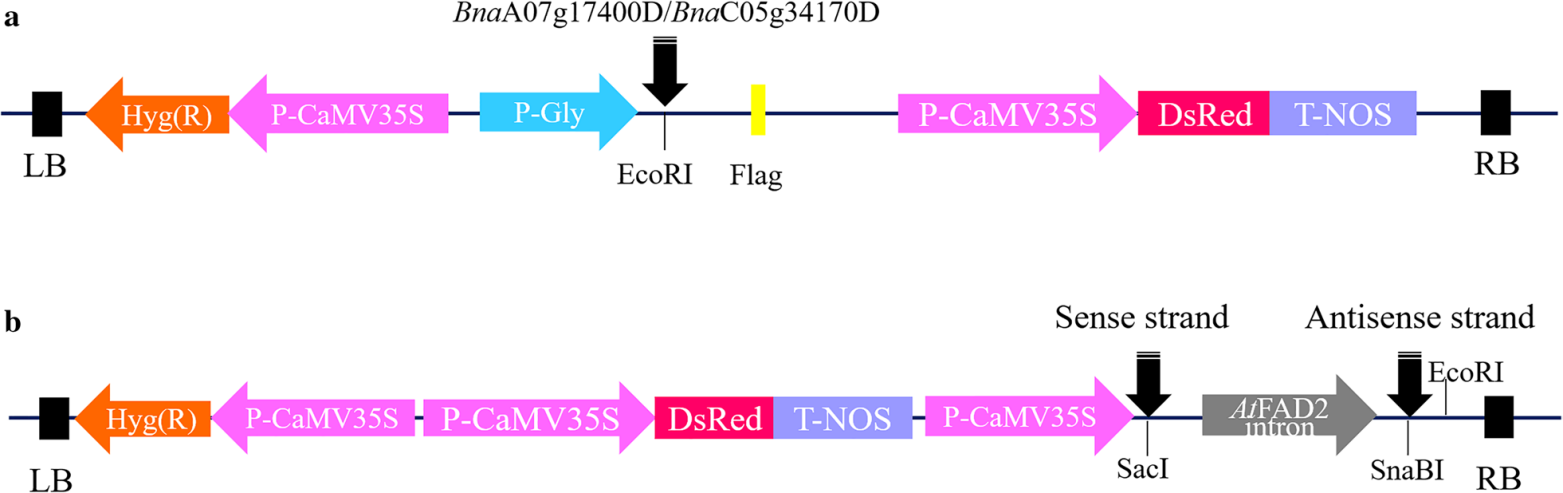
Vectors construction for gene over-expression and knock-down. **a** Diagram of the construction of the over-expression vector. The expression of *Bna*A07g17400D and *Bna*C05g34170D is driven by the glycinin promoter. The N-terminus of *Bna*A07g17400D and *Bna*C05g34170D is accompanied by a flag. **b** Diagram of the construction of the RNAi knock-down vector. The expression of sense strands and antisense strands is driven by the 2 × 35S promoter and a 2 × nuclear localization signal (NLS) at the N-terminus. *DsRed* in both vectors was also under the control of the CaMV35S promoter

For *Agrobacterium*-mediated hypocotyl transformation in rapeseed, the over-expression vector pCMABIA-1303 and RNAi vector p35S-1390 were used for transformation (Fig. 1). Consequently, two over-expression vectors were constructed for *Bna*A07g17400D and *Bna*C05g34170D (hereafter called E1 and E2), respectively. Additionally, three RNAi vectors were also designed and constructed to knock down these two genes, i.e., two vectors were for *Bna*A07g17400D (hereafter called R1 and R2) while the third one was for *Bna*C05g34170D (hereafter called R3). In E1 and E2, the expression of *Bna*A07g17400D and *Bna*C05g34170D was controlled by the glycinin promoter, while in R1, R2 and R3, sense and antisense strands of RNAi fragments were guided by the CaMV35S promoter (Fig. 1). The marker gene-*DsRed*, was also under the control of the CaMV35S promoter (Fig. 1).

Hypocotyls from eight different rapeseed varieties were used for *Agrobacterium* mediated transformation. The formation from calli to regenerated plantlets on a hypocotyl, i.e., calli formation, shoots formation, roots formation and plantlet regeneration, showed a good condition during the regeneration process (Additional file 1). It is known that the T-DNA integration ability into the plant genome is an important factor for plant transformation. Thus, the concentration of *Agrobacterium* was carefully controlled in this investigation. To simplify the final concentration adjustment, only the original OD_600_ needs to be examined for further experiments. Thus, 2 ml *Agrobacterium* solution at the original concentration was collectedat OD_600_ = 0.4 to suspended in 2 ml LIM and then diluted to 20 ml for infection. The original OD_600_ was sometimes higher than 0.4, and it might have reached up to 1.2 after inoculation. In this case, pipette approximately (0.4/OD_600_) * 2 ml *Agrobacterium* solution to collect the pellet. For example, pipette 1.5 ml at OD_600_ approximately 0.6 to collect the pellet, and finally suspended it in 2 ml LIM for further infection. Using this strategy, the final OD_600_ was approximately 0.04 for hypocotyl infection. In addition, 2,4-D was also used for cocultivation during calli induction to facilitate the integration of foreign DNA. Additionally, the ability of the transgenic calli to develop into a complete plant was also vital for plant regeneration. Thus, STS, which consisted of 0.1 M Na_2_S_2_O_3_ and 0.1 M AgNO_3_ at a 4:1 ratio, was added to the CIM to avoid tissue browning, while 3 mg/L AgNO_3_ was added to the SIM to promote shoot regeneration. Finally, 1 mg/L IBA was added to root formation medium for root generation (detailed methods in “Materials and methods” section). In short, eight different rapeseed varieties were separately transformed via the aforementioned *Agrobacterium*-mediated hypocotyl transformation in *B. napus*.

### *DsRed* for rapid and convenient screening during tissue culture

**Fig. 2:**
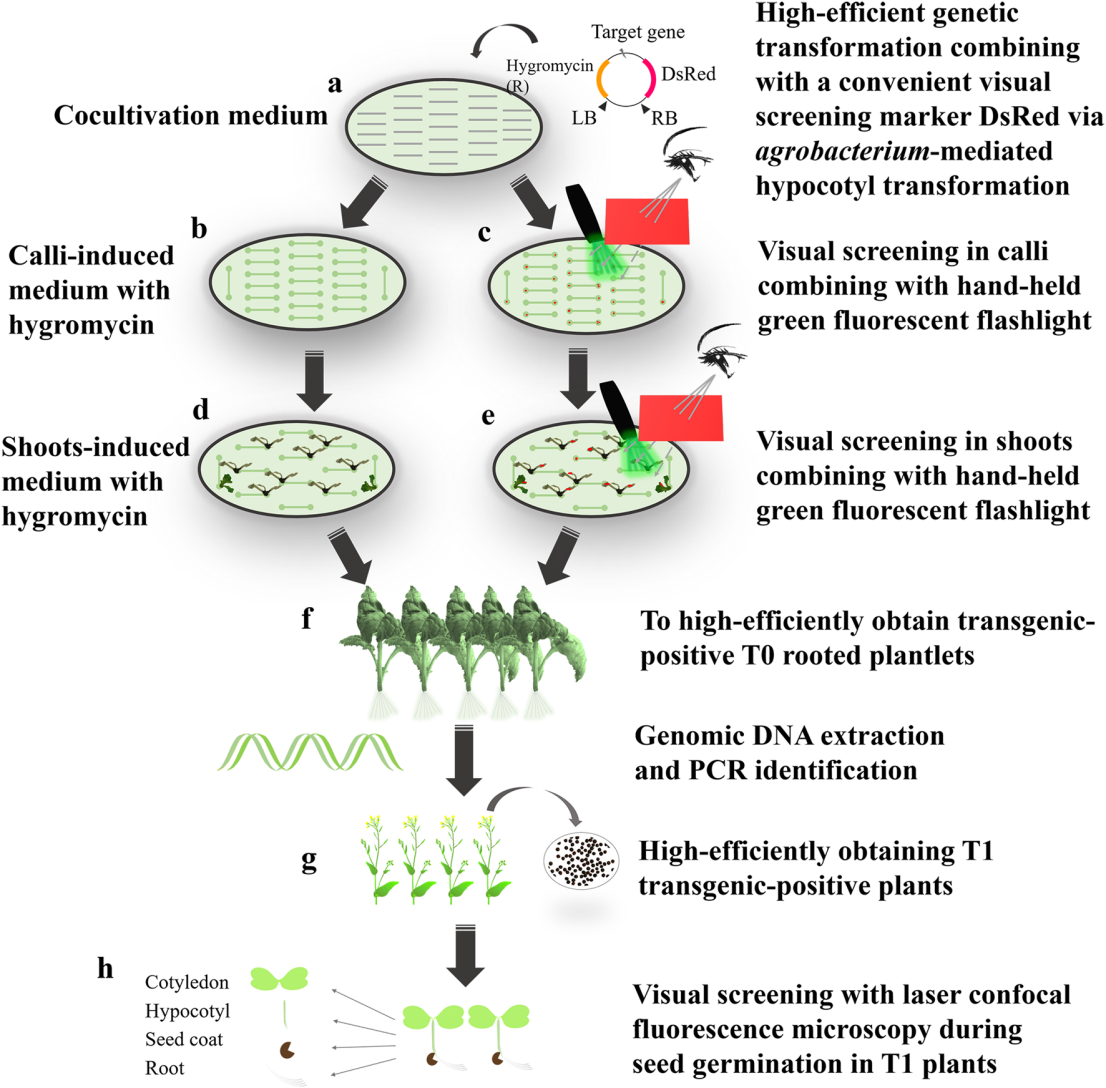
Overview of the efficient method for the high-efficiency acquisition of transgenic lines combined with a visual screening method compared with antibiotics screening in *B. napus*. **a** Highly efficient genetic transformation combined with a convenient visual screening marker *DsRed* via *Agrobacterium*-mediated hypocotyl transformation in *B. napus*. Hypocotyls were transformed with a vector accompanied by *DsRed* and hygromycin via *Agrobacterium*- mediated transformation. The light grey lines inside the oval represent hypocotyls from *B. napus*. **b** , **c** Calli formation and screening during the callus-induced stage with or without the visual screening method. The calli would survive under antibiotics screening with hygromycin in **b**, and they were further picked by the visual screening using a hand-held green fluorescent flashlight in **c**. The endpoints in the line represent the formed callus, and the red spots in the endpoints represented the red fluorescence observed in the calli. **d** ,**e** Shoots formation in shoot-induced medium with or without the visual screening method. The calli would survive under antibiotics screening with hygromycin in d, and they were further picked for visual screening using a hand-held green fluorescent flashlight in e. The red spots in the shoots represent the red fluorescence observed in the cotyledons. **f**, **g** highly efficient screening of the transgenic-positive rooted T0 plantlets and T1 transgenic-positive plants. Transgenic-positive rooted T0 plantlets and T1 transgenic-positive plants could be rapidly and high efficiently obtained when combined with the convenient visual screening method. **h** Visual screening in cotyledons, hypocotyls, seed coats and roots during seed germination. The seedlings could be screened with the visual marker *DsRed* using laser confocal fluorescence microscopy

**Fig. 3:**
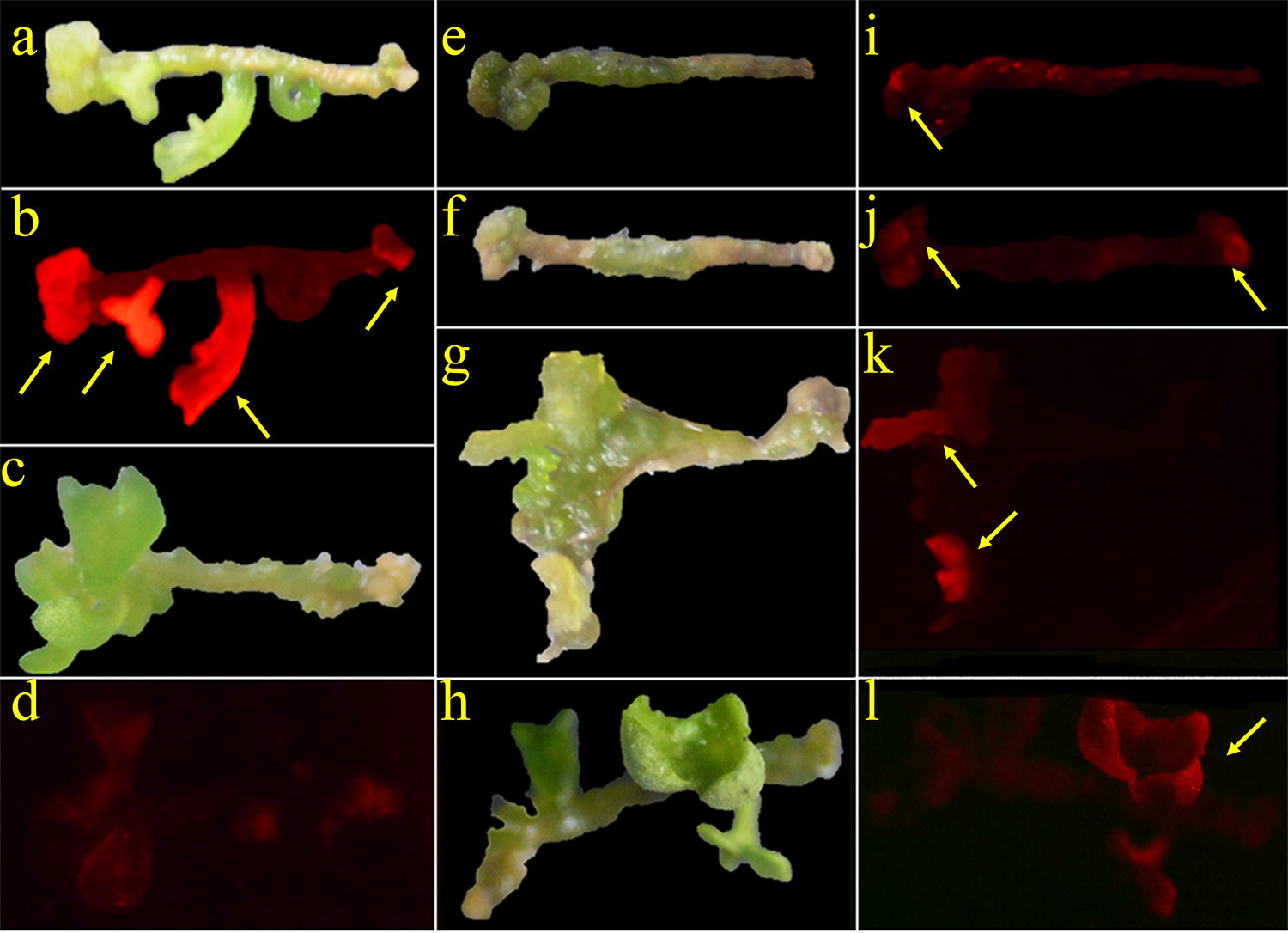
Efficient and convenient screening in the primary stage of tissue culture. **a**, **b** Examples of positive tissue with red fluorescence by visual screening. Strong red fluorescence could be observed in calli and shoots. **c**, **d** Examples of negative tissue with red fluorescence by visual screening. Nearly no or weak red fluorescence in calli and shoots could be observed. **e**–**l** Diagram of the convenient screening for calli and shoots at the primary culturing stage. Red fluorescence could be clearly observed in calli and shoots by visual screening with a hand-held green fluorescent flashlight. The yellow arrows indicate red fluorescence. **a**, **c**, **e**–**h** are in the white field, while **b**, **d**, **i**, **g**, **k** and **l** are in the excitatory light field

Considering that the expression of *DsRed* occurred in the whole plant development process, it is presumed that the red fluorescence present in transgenic tissue could be observed through a red filter with a hand-held green fluorescent flashlight. Therefore, the red fluorescence emitted by *DsRed* might be utilized to screen transgenic-positive tissues in the primary stage of tissue culture. In this way, it was attempted to distinguish the transgenic-positive and transgenic-negative calli and shoots in tissue culture medium. As expected, strong red fluorescence was observed in transgenic-positive calli and shoots (Fig. 3a, b), while nearly no or weaker fluorescence could be observed in transgenic-negative ones (Fig. 3c, d). Moreover, red fluorescence emitted by putative transgenic-positive calli or shoots could also be clearly distinguished under a fluorescence stereomicroscope (Additional file 2). This strong contrast indicated the feasibility of using *DsRed* as a visual screening marker in tissue culture of *B. napus*. Thus, plenty of transformed rapeseed hypocotyls, containing calli and shoots, were observed with red fluorescence on CIM and SIM (Figs. 2b–e and 3e–i). Using this method, the transgenic-positive and transgenic-negative shoots could be clearly distinguished, demonstrating the feasibility of screening positive regenerative plantlets conveniently with a visual screening method. Furthermore, the calli, which could be observed with red fluorescence, were marked and counted under a hand-held green fluorescent flashlight. The calli with red fluorescence ranged from 50.8 to 71.4%, i.e., 56.9% (78/137) for E1, 50.6% (66/131) for E2, 66.9% (73/109) for R1, 50.8% (97/191) for R2 and 71.4% (167/234) for R3 (Table 1). These results showed that the average transformation efficiency was achieved to 59.1%, and they revealed that the visual screening method by red fluorescence provided an obvious advantage for rapid identification and convenient discrimination during the early stage of tissue culture.

**Table 1:** Transformation efficiency of red fluorescence in different lines

Target gene	Number of experiments	Number of hypocotyls inoculated	Hypocotyls with *DsRed*	Transformation efficiency (%)
*Bna*A07g17400D	1	77	47	61.0
	2	60	31	51.7
*Bna*C05g34170D	1	57	30	52.6
	2	74	36	48.6
R1	1	59	35	59.3
	2	50	38	76.0
R2	1	92	47	51.1
	2	99	50	50.5
R3	1	143	109	76.2
	2	91	58	63.7

### *DsRed* for efficient genetic transformation during tissue culture

To investigate whether the calli that emitted red fluorescence were integrated with the desired objective DNA fragments, the potential transgenic calli and shoots were randomly selected for identification by PCR amplification. Hypocotyls of three commercial rapeseed varieties (Zhong shuang 8, Zhong shuang 11, Zhong you 821), a spring rapeseed variety (Jia 9709) and a semi-winter variety (Jia 2016) were transformed with R2 vector. As expected, almost all the commercial cultivars carried the *DsRed* amplification products, i.e., 95.5% (21/22), 100% (22/22), 95.5% (21/22), 100% (22/22) and 100% (22/22) were identified with PCR products having a length of 426 bp (Additional file 3a). This high-efficiency genetic transformation system showed that it was a successful attempt in different rapeseed varieties with visual screening showing red fluorescence from the induced calli to shoots.

Additionally, to further identify whether this transformation system was also appropriate for other rapeseed varieties with different expression vectors, hypocotyls from another three commercial rapeseed varieties (7633, B 351 and Shan 3B), which were transformed with E1 vector, were also analysed by PCR detection. The results showed that 83.3% (15/18), 94.4% (17/18) and 100% (18/18) were amplified at the length of 722 bp for the *Bna*A07g17400D gene (Additional file 3b). Consequently, these results showed that the different commercial rapeseed varieties and different expression vectors used in this study were competent for transformation using the *Agrobacterium*-mediated hypocotyl transformation method (Fig. 2). Thus, an average of 96.1% of the tissues observed with red fluorescence could indeed be checked with the objective gene in eight different rapeseed varieties. These results verified that the visual screening method with *DsRed* could be used to shorten the screening period and accelerate the regeneration process to obtain high-efficiency positive transgenic plantlets.

### High-efficiency for rooted transgenic-positive plantlets in rapeseed

To investigate the transgenic-positive efficiency via the visual screening method based on *Agrobacterium*-mediated transformation in rapeseed, plantlets transformed with E1, E2, R1, R2 and R3 vectors that were regenerated from hypocotyls of “Jia 2016” were collected, respectively. Approximately 196 independent rooted transgenic plantlets were obtained, and all these positive transgenic plants had a normal phenotype both in vegetative growth and reproductive growth; no sterile transgenic plants were found in the field.

**Fig. 4:**
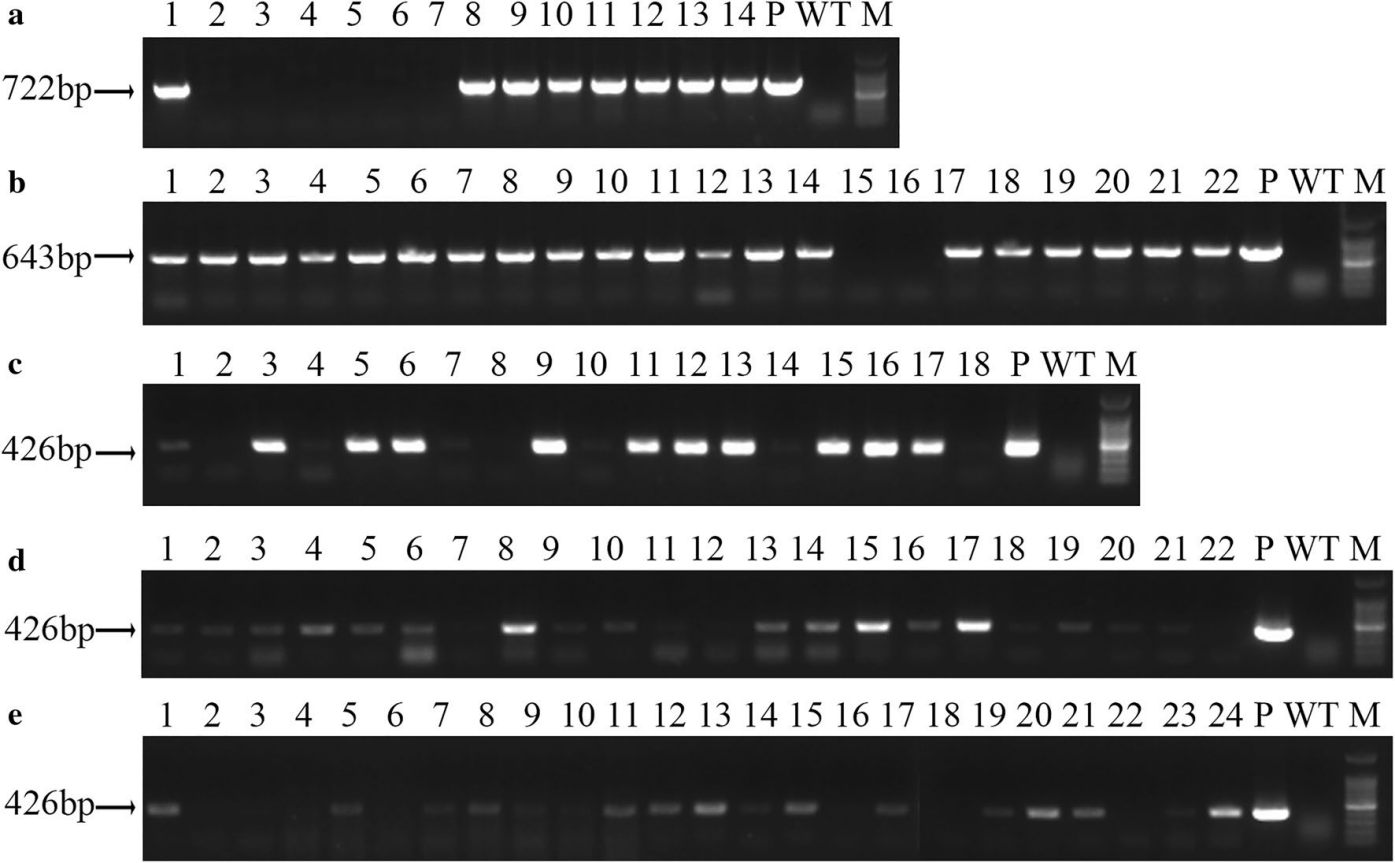
Transgenic-positive plant identification in T0 plants. **a** Amplification of the *BnaA07g17400D* gene in E1 transformed lines. **b** Amplification of the *BnaC05g34170D* gene in E2 transformed lines. **c**–**e** Amplification of *DsRed* gene in R1, R2 and R3 transformed lines. The light bands indicated by the arrow indicate the size of the amplified genes noted on the left side. P, plasmid. WT, wild type plant. Marker, DL 100 bp ladder

Next, genomic DNA was extracted from putative transgenic rapeseed plantlets, and PCR amplification was performed to detect the integration of *DsRed*, *Bna*A07g17400D and *Bna*C05g34170D. As expected, most of the putative transgenic lines was presented positive bands on the agarose gel (Fig. 4). To be specific, 80% (8/10) and 90.7% (49/54) were detected for *Bna*A07g17400D and *Bna*C05g34170D amplification, respectively (Table 2). In addition, 88% (22/25), 81.4% (35/43) and 89.1% (57/64) were detected for R1, R2 and R3 by *DsRed* fragment amplification, respectively (Table 2). Hence, five different expression vectors, including over-expression and RNAi-mediated knock-down, could be efficiently transformed into rapeseed with the *Agrobacterium*-mediated hypocotyl transformation system.

**Table 2:** Transformation efficiency using different expression vectors in J2016

	Total	Transgenic positive	Transformation efficiency (%)
*Bna*A07g17400D	10	8	80.0
*Bna*C05g34170D	54	49	90.7
R1	25	22	88.0
R2	43	35	81.4
R3	64	57	89.1

### *DsRed* for convenient and efficient screening during seed germination

**Fig. 5:**
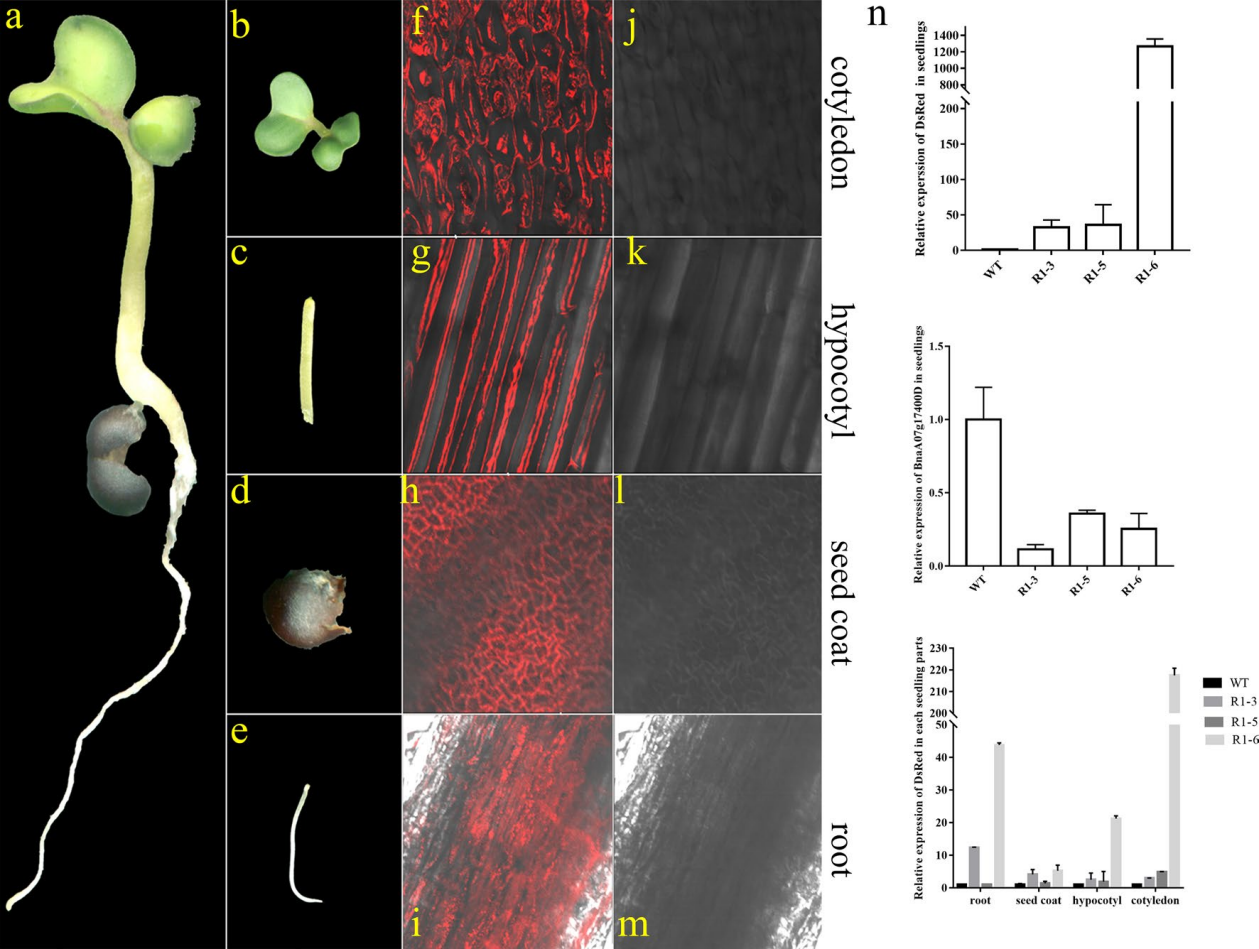
Convenient screening during seed germination. **a**–**m** Observation of red fluorescence through the primary stage of seedling. The red fluorescence could be observed inthe cotyledon (**b**, **f**, **j**), hypocotyl (**c**, **g**, **k**), seed coat (**d**, **h**, **l**) and root (**e**, **i**, **m**) of rapeseed seedlings under a laser confocal fluorescence microscope FV1000, respectively. **n** RT-PCR identification of the relative gene expression in seedlings. The upper diagram represents the relative gene expression of *DsRed* in seedlings from different RNAi lines. The middle diagram represents the relative gene expression of BnaA07g17400D in seedlings from different RNAi lines. The lower diagram represents the relative gene expression of *DsRed* in roots, seed coats, hypocotyls and cotyledons from seedlings of different RNAi lines

Considering the thick tissue in *B. napus*, it is not easy to distinguish the positive transgenic seeds and seedlings with a hand-held green fluorescent flashlight after obtaining T1 seeds and regeneration plants. Red fluorescence, unlike green fluorescence, can be observed under laser confocal fluorescence microscopy without the interference of chlorophyll. To verify the screening feasibility during the primary stage of seed germination, experiments were performed using a confocal microscope. For each part, seed coats, roots, hypocotyls and cotyledons, were randomly selected for further analysis (Figs. 2h and 5a–e). It was revealed that the red fluorescence could be successfully observed in all the cells of different parts of the seedings (Fig. 5f–m).

Furthermore, RT-PCR analysis was performed to check the relative expression of the marker gene—*DsRed* and the target gene—*Bna*A07g17400D in the seedlings during seed germination (Fig. 5n). As expected, the exogenous marker gene—*DsRed* showed significant up-regulation in different transgenic lines, and achieved to even 1200 times higher levels compared with WT. Regarding the inferenced endogenous gene, the relative gene expression of *Bna*A07g17400D showed obvious down-regulation in the transgenic seedlings from different RNAi lines. Additionally, in the roots, seed coats, hypocotyls and cotyledons of seedlings from the aforementioned RNAi lines, the relative expressions of *DsRed* presented a comparable up-regulation in various organs, especially in cotyledons, which indicated that red fluorescence could be more easily detected in cotyledons. In brief, *DsRed*, as a marker gene, is reliable for positive screening without influencing the desired RNAi events.

### Gene copy number identification of *DsRed* by southern blotting

**Fig. 6:**
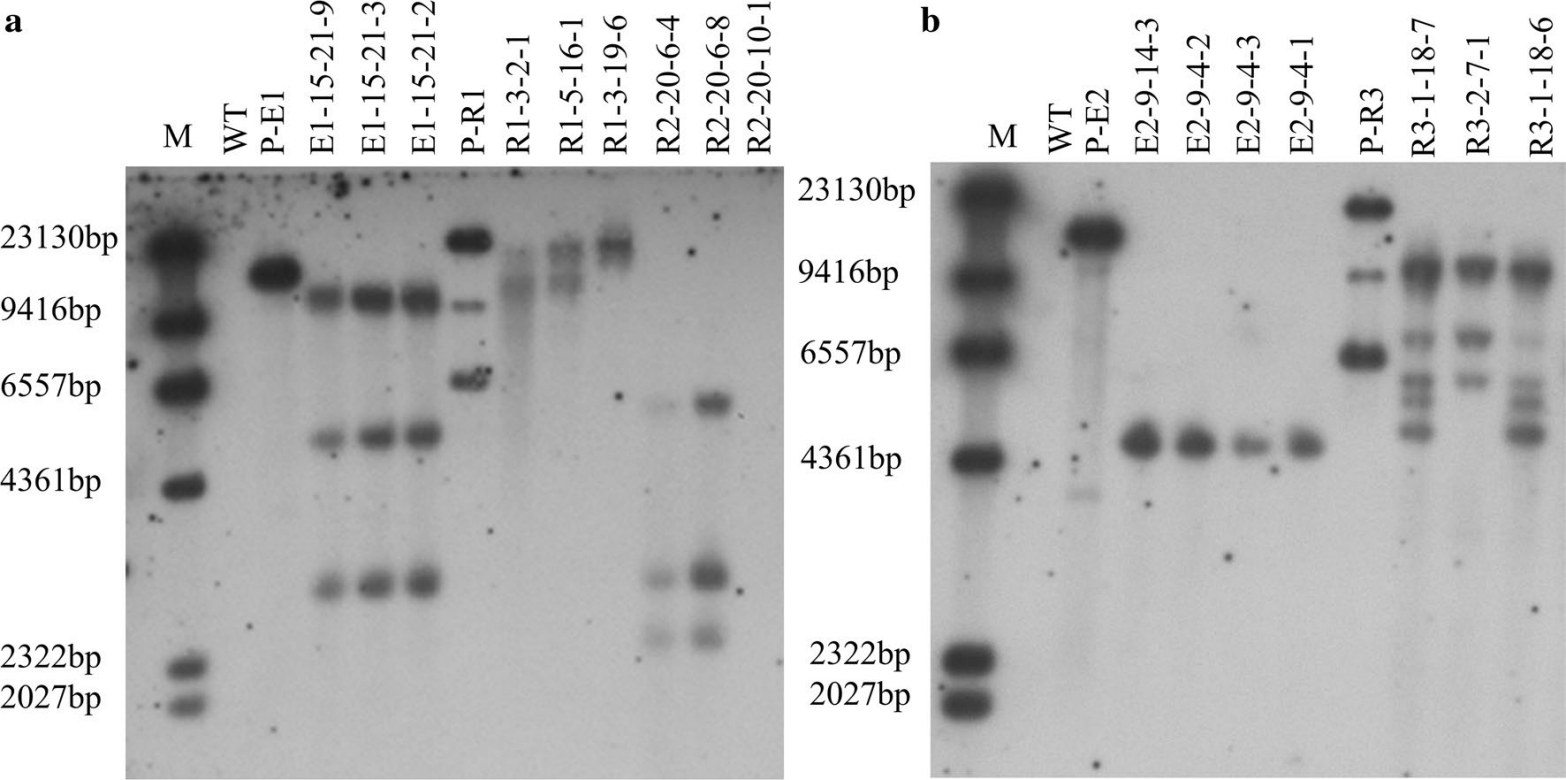
Southern blot analysis of *DsRed* genes for copy number identification in T3 transgenic rapeseed plants. **a** Southern blot analysis of *Bna*A07g17400D over-expression and knock down. **b** Southern blot analysis of *Bna*C05g34170D over-expression and knock down. WT, wild type; P-E1/E2, over-expression plasmid pCMABIA-1303; P-R1/R2/R3, knock down plasmid p35S-1390; M: λ DNA/Hind III- Plus DNA marker

To verify whether *DsRed* could be applied to identify the integrated copy number in regenerated plants, Southern blotting was used to detect the integration of *DsRed* cassettes in several transgenic plants. Three T3 plants for each line were randomly chosen for Southern blotting, and eight were confirmed to contain the insertion of the *DsRed* gene (Fig. 6). Southern blotting results revealed that the *DsRed* gene showed one—to—five copies in the transgenic rapeseed plants. More specifically, in the E1 lines, all three plants were inserted with three copies. In the R1 lines, one plant was inserted with a single copy and two plants with two copies. In the R2 lines, two plants were inserted with three copies, and the third plant lacked an insertion, which indicated that R2-20–10-1 was a marker-free transgenic plant (Fig. 6a). In the E2 lines, all three plants carried a single copy. In the R3 lines, two plants had five copies and one plant had three copies (Fig. 6b).

It is worth noting that all E1 lines that stemmed from the same parent (E1-15–21) presented the same three T-DNA copies (Fig. 6a). Similarly, all the E2 lines from one T1 plant (E2-9) had one copy, and the two R2 lines from the same T2 plants (R2-20–6) also contained three T-DNA copies (Fig. 6a). These findings indicated that E1-15-21, R2-20-6 and E2-9 could be regarded as stable and homozygous transgenic plants. However, in R1 and R3 lines, the T-DNA copy number showed diversity in different plants), and the copy number of the third R2 plant (R2-20-10-1) also differed from the previous two plants because of the different parents (Fig. 6a). These results demonstrated that the T-DNA copy numbers were invariable between the same T2 lines (E1-15-21 and R2-20-6) but variable among the different T2 lines (R1-3-2, R1-3-19, R1-5-19 and R2-20-10), illustrating that stable transgenic plants could be obtained in T3 plant, even in tetraploid rapeseed plants.

## Discussion

Over the last century, many attempts have been conducted in *Agrobacterium*-mediated transformation of rapeseed [5, 41–44]. However, few studies have been focused on the concentration of *Agrobacterium*. Previously, the concentration for *Agrobacterium* infection was controlled by adjusting the final OD_600_ [5]. Here, not the final OD_600_ adjustment but the calculation of the volume from the original *Agrobacterium* solution was performed, and different commercial rapeseed varieties, different expression vectors and different genes were transformed efficiently in this way. It is noteworthy that the OD_600_ was only detected once for the inoculated *Agrobacterium*, and the pipette volume for further experiments could be easily calculated by the aforementioned method. Previously, the final OD_600_ was adjusted to a fixed value in other studies, such as 0.05, 0.2 or 1, based on the original OD_600_ for explants infection [25, 26, 43]. However, the original OD_600_ usually ranged from 0.4 to 1.2 in this investigation, owning to the differences in the original inoculum, bacterial concentration, and bacterial activity. Considering the sensitivity of explant to the *Agrobacterium*, the final concentration was found to be easily controlled by the volume of the original *Agrobacterium* solution rather than an adjustment to avoid explants turning necrotic. Therefore, ensuring the appropriate bacterial activity of collected *Agrobacterium* was of great significance. Moreover, the timing of incubation infection and cocultivation stage should be carefully controlled, as excessive attachment can directly lead to explant necrosis or reduced regeneration [5, 45]. Usually, 10 min was used for hypocotyls infection, with maintenance for 36–48 h in the cocultivation medium in this investigation. Sometimes, the time for infection could be extended or shortened by 1–2 min appropriately and flexibly if the collected *Agrobacterium* or the hypocotyls were not in good condition. After 24 h of cocultivation in cocultivation medium, the hypocotyls should be tough and bright white, which can be transferred to CIM 24 h later. If the hypocotyls do not show the aforementioned features, it was suggested that they should be transferred to the CIM immediately or up to 12 h later to avoid explant necrosis. In this way, highly efficient transformation was obtained in eight rapeseed varieties. Previously, 2,4-D was reported to be added to the cocultivation medium to facilitate the integration of foreign DNA, owing to its ability to enable cell division at the incision [46]. Additionally, AgNO_3_ is known to promote the generation and elongation of shoot primordia, and increase the frequency of adventitious shoot differentiation [42]. Thus, an average of 85.8% transgenic-positive rooted plantlets is obtained with the addition of 1 mg/L 2,4-D to CIM and 3 mg/L AgNO_3_ to SIM. No obvious difference was observed in the transformation efficiency among different vectors, except for E1, which was utilized in our exploratory study for optimum experimental conditions. Therefore, the establishment of an efficient *Agrobacterium*-mediated rapeseed transformation system using hypocotyls will also enable genome editing of valuable genes controlling important traits.

Based on the well-established transformation system with high transformation efficiency, an effective transgenic-positive screening technology is of great importance for shortening the period of tissue culture and simplifying the transformation system in rapeseed (Fig. 2). *DsRed*, as a selectable marker gene for transgenic seed screening, is advantageous for separating transgenic and non-transgenic seeds in many plants, such as *Arabidopsis*, rice and *Camelina sativa* [15, 47, 48]. In *Arabidopsis*, *DsRed* has been used as a visual selection marker to promote transgenic seed screening [33, 49, 50]. However, considering the impediment of tissue thickness, *DsRed* has not been used in rapeseed for convenient screening to accelerate the process of transgenic-positive plantlet selection.

The red fluorescence emitted by *DsRed* marked calli and shoots could be easily observed through a red filter using a hand-held green fluorescent flashlight. Up to 61.2% of the calli were observed with red fluorescence, and almost all of these calli could be detected with the *DsRed* gene by PCR analysis. The red fluorescence in calli and rapeseed seedlings could also be observed under a fluorescence stereomicroscope and laser scanning confocal microscope, respectively. Moreover, it is worth noting that the fluorescence intensities varied from each other, potentially due to the rapeseed genotypes, i.e., homo- or hemizygosity of the transgene [50]. Additionally, *DsRed*, as a marker for Southern blotting analysis, was also successfully applied for estimating one to five copies in the transgenic offspring plants, as in other studies [41, 42]. These results indicated that the visual screening marker *DsRed* is a great success in accelerating the genetic transformation system in *B. napus*.

In addition, several methods provide alternatives for screening transgenic positive plants (Fig. 2 and Table 3). Antibiotic screening is frequently used in most transformation studies, such as kanamycin, hygromycin or herbicide [26, 51, 52]. However, the dosage of these substances might influence the regeneration rate and further break the balance of transgenic/non-transgenic seeds. PCR amplification is time and cost consuming in plant preparation and identification. Southern blot analysis, as a traditional method for identification of the transgenic copy number, is costly in terms of reagents, equipment, time and labour [40]. The GUS expression assay is a compromise in the matter of cost. However, it usually requires a long time for tissue decolouring. Moreover, the GUS expression assay is often performed following the expression of target gene in plant cells or tissues [53]. As for GFP, it is usually used for determining sub-cellular localization with a laser confocal fluorescence microscope, which should be associated with RFP or YFP due to its instability [54, 55]. Otherwise, it can also be detected with a fluorescence microscope and a green fluorescence pigment filter using tissue stained with fluorescein di-acetate (FDA), which induces a decreased fluorescence intensity [54, 56]. Nevertheless, the FDA assay has hardly used in plants to our limited knowledge. Compared with previous methods, the present study is the first to introduce the visual selectable marker gene *DsRed* to accelerate transgenic plantlets screening. Only a combination of light filter with hand-held fluorescent flashlight to easily distinguish the transgenic-positive and transgenic-negative plants during the primary stage of tissue culture and seed germination in rapeseed. Therefore, the application of *DsRed*, which served as a marker gene, will facilitate the screening of transgenic positive lines and the utilization of tissue culture or cell culture in plant biotechnology.

**Table 3:** Comparisons of different screening methods in plants

Methods	Special reagents	Equipments	Total time (D) /1000 samples	Cost/200 reaction	Labour	Injuries to plant
PCR amplification	DNA extarct kit	Thermal cycler	2	High	High	Leaf
Antibiotic/Herbicide selection	Autobiotics	–	≥ 2	Low	Moderate	Seed
Southern blot analysis	Southern blot hybridization kit	Hybridisation oven	≥ 7	High	High	Leaf
Gus expression assays	GUS dye	Microscopy	≥ 2	Moderate	Moderate	Leaf/Root
GFP	Fluorescein di-acetate^a^	Laser scanning confocal microscopy/green fluorescence pigment	≥ 0.5	Moderate	Moderate	Leaf/Root
Visual screening	No	Hand-held fluorescent flashlight, light filter	≤ 0.5	Zero	Low	No

^a^It has mostly been used in microorganisms

## Conclusion

A convenient and highly efficient method was developed that combines an efficient genetic transformation system with a convenient and rapid transgenic-positive visual screening method in rapeseed. Using this method, potential transgenic-positive plants could be quickly and efficiently selected by a convenient visual screening method, which greatly accelerated the regeneration process. Additionally, a high transgenic positive efficiency could be easily achieved based on the optimized *Agrobacterium*-mediated hypocotyl transformation, which greatly increased the number of genetic transformed plants. The aforementioned system will be a good alternative for rapeseed genetic transformation and gene functional studies.

## Supplementary information

**Additional file 1.** Formation of calli to a regenerated plantlet on a hypocotyl.** a** and** b** calli that were induced from hypocotyls.** c** Calli that were prepared for swollen to shoots.** d** and** e** Shoot formation and cotyledon development accompanied by the appearance of a growth point.

**Additional file 2.** Visual screening of transgenic-positive and/or -negative tissues under a fluorescence stereomicroscope. Red fluorescence could be observed under fluorescence stereomicroscope to facilitate visual screening in tissue culture medium. The image was captured in bright field **a** and its corresponding dark field **b**, respectively. The tissues indicated by the arrow represent transgenic-positive calli.

**Additional file 3.** PCR amplification for transgenic-positive identification in calli.** a** Amplification of *DsRed* from calli of Jia 9709, Jia 2016, Zhong shuang 8, Zhong shuang 11 and Zhong you 821, respectively. P, plasmid. WT, wild type plant. Marker, DL 100 bp ladder. **b** Amplification of *Bna*A07g17400D from calli in 7633, B 351 and Shan 3B, respectively. P, plasmid. N, wild type plant. Marker, DL 2000 bp.

**Additional file 4.** Primers used in this study.
