# Supplementary material for: A convenient, rapid and efficient method for establishing transgenic lines of Brassica napus
Source: Plant Methods. 2020 Mar 30;16:43. doi: 10.1186/s13007-020-00585-6 (PMC7106750; doi:10.1186/s13007-020-00585-6)
Supplement: Supplementary file 1 — Additional file 1. Formation of calli to a regenerated plantlet on a hypocotyl. a and b calli that were induced from hypocotyls. c Calli that were prepared for swollen to shoots. d and e Shoot formation and cotyledon development accompanied by the appearance of a growth point. [file 13007_2020_585_MOESM1_ESM.doc]

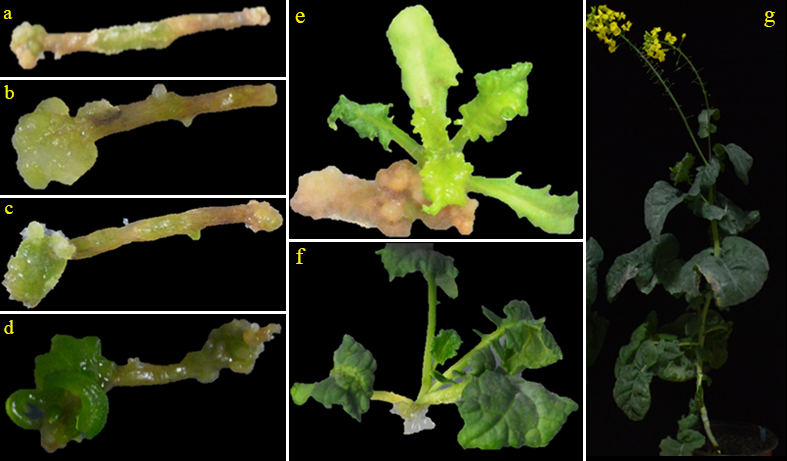


**Additional file 1.** **Formation of calli to a regenerated plantlet on a hypocotyl.** **a** and **b** calli that were induced from hypocotyls. **c** Calli that were prepared for swollen to shoots. **d** and **e** Shoot formation and cotyledon development accompanied by the appearance of a growth point.
