# Supplementary material for: A convenient, rapid and efficient method for establishing transgenic lines of Brassica napus
Source: Plant Methods. 2020 Mar 30;16:43. doi: 10.1186/s13007-020-00585-6 (PMC7106750; doi:10.1186/s13007-020-00585-6)
Supplement: Supplementary file 2 — Additional file 2. Visual screening of transgenic-positive and/or -negative tissues under a fluorescence stereomicroscope. Red fluorescence could be observed under fluorescence stereomicroscope to facilitate visual screening in tissue culture medium. The image was captured in bright field a and its corresponding dark field b, respectively. The tissues indicated by the arrow represent transgenic-positive calli. [file 13007_2020_585_MOESM2_ESM.doc]

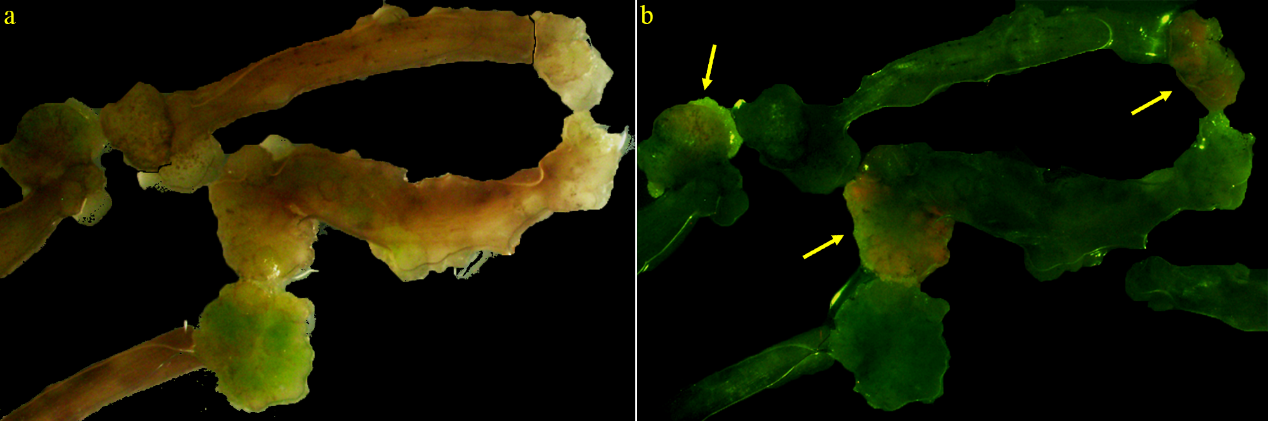


**Additional file 2.** **Visual screening of transgenic-positive and/or -negative tissues under a fluorescence stereomicroscope.** Red fluorescence could be observed under fluorescence stereomicroscope to facilitate visual screening in tissue culture medium. The image was captured in bright field **a** and its corresponding dark field **b**, respectively. The tissues indicated by the arrow represent transgenic-positive calli.
