# Supplementary material for: A convenient, rapid and efficient method for establishing transgenic lines of Brassica napus
Source: Plant Methods. 2020 Mar 30;16:43. doi: 10.1186/s13007-020-00585-6 (PMC7106750; doi:10.1186/s13007-020-00585-6)
Supplement: Supplementary file 3 — Additional file 3. PCR amplification for transgenic-positive identification in calli. a Amplification of DsRed from calli of Jia 9709, Jia 2016, Zhong shuang 8, Zhong shuang 11 and Zhong you 821, respectively. P, plasmid. WT, wild type plant. Marker, DL 100 bp ladder. b Amplification of BnaA07g17400D from calli in 7633, B 351 and Shan 3B, respectively. P, plasmid. N, wild type plant. Marker, DL 2000 bp. [file 13007_2020_585_MOESM3_ESM.doc]

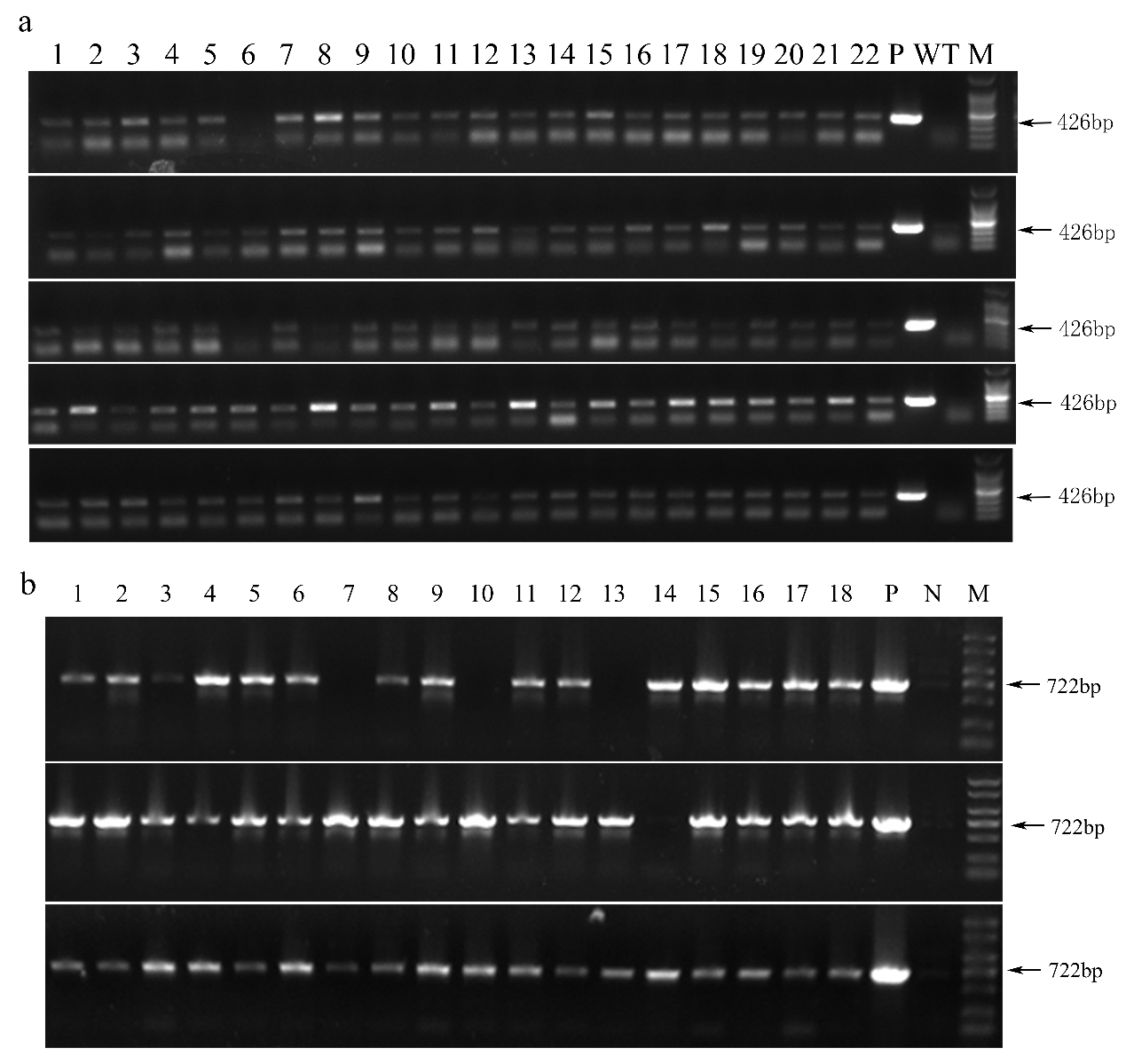


**Additional file 3.** **PCR amplification for transgenic-positive identification in calli.** **a** Amplification of *DsRed* from calli of Jia 9709, Jia 2016, Zhong shuang 8, Zhong shuang 11 and Zhong you 821, respectively. P, plasmid. WT, wild type plant. Marker, DL 100bp ladder. **b** Amplification of *Bna*A07g17400D from calli in 7633, B 351 and Shan 3B, respectively. P, plasmid. N, wild type plant. Marker, DL 2000bp.
