# Supplementary material for: A convenient, rapid and efficient method for establishing transgenic lines of Brassica napus
Source: Plant Methods. 2020 Mar 30;16:43. doi: 10.1186/s13007-020-00585-6 (PMC7106750; doi:10.1186/s13007-020-00585-6)
Supplement: Supplementary file 4 — Additional file 4. Primers used in this study. [file 13007_2020_585_MOESM4_ESM.doc]

| **Additional file 4. Primers Used in This Study** | | |
| --- | --- | --- |
| R1-S-F | 5’ CCGGTACCAGGCCTGAAATTTCTCCCAGTCATA | RNAi vector construction |
| R1-S-R | 5’ ACGTAGGGGCGATAGAGTGCATCCTTAGTCA 3’ |  |
| R1-A-F | 5’ TGGAAGACGCGTTACAGTGCATCCTTAGTCA 3’ |  |
| R1-A-R | 5’ GGATCCGTCGACTACAAATTTCTCCCAGTCATA 3’ |  |
| R2-S-F | 5’ CCGGTACCAGGCCTGTGCCTGTCCCTCGAAA 3’ |  |
| R2-S-R | 5’ ACGTAGGGGCGATAGAAGAGATTTGACCAGTGT 3’ |  |
| R2-A-F | 5’ TGGAAGACGCGTTACTTACTAACAGCTGACACA 3’ |  |
| R2-A-R | 5’ GGATCCGTCGACTACCATGCTCTTGTCGTTT 3’ |  |
| R3-S-F | 5’ CCGGTACCAGGCCTGTGGATGTACTTCTGGGATC 3’ |  |
| R3-S-R | 5’ ACGTAGGGGCGATAGTGATTCGACGGATGTGG 3' |  |
| R3-A-F | 5’ TGGAAGACGCGTTACTGATTCGACGGATGTGG 3’ |  |
| R3-A-R | 5’ GGATCCGTCGACTACTGGATGTACTTCTGGGATC 3’ |  |
| DsRed-F | 5'CCGGCGCGCCAAGCTCTAGTAGAAGGTAATTATCCA3' |  |
| DsRed-R | 5'GTAGGGAGCTAAGCTCCCGATCTAGTAACATAGAT3' |  |
| BnaA07g17400D | 5'CCGCGGCCGCGAATTCATGGCGATGGCAGCAGCA3' | over-expression vector construction |
|  | 5'CCTTGTAATCGAATTCCTTCTGCTTCTCCTCCAC3' |  |
| BnaC05g34170D | 5'CCGCGGCCGCGAATTCATGGAAAAGAAACTTACAAACT3' |  |
|  | 5'CCTTGTAATCGAATTCAAAGTTCATTTTTTTACTAATTTG3' |  |
| Actin | F: AGAGTCATGCCAAGTTCATGGTT | RT-PCR |
|  | R: CCTCATAAGCACACCATCAACTCTAA |  |
| BnaA07g17400D-RT | F: 5' AGAGGCAAAACTTAAAGCAGCACA 3' |  |
|  | R: 5' TTTGGTGCGAGGAATCAACACATT 3' |  |
| DsRed-RT | F: 5' CCCGCCGACATCCCCGACT 3' |  |
|  | R: 5' CGAAGTTCATCACGCGCTCCC 3' |  |
| BnaA07g17400D-OE | F: 5' TAAAACACTTACAACACCGGAT 3' | PCR identification |
|  | R: 5' GAGGACATAGCAAACTGCC 3' |  |
| DsRed-OE | F: 5' GCCAAGCTCTAGTAGAAGGT 3' |  |
|  | R : 5' TATATAGGAAAACTCAAGGGCAAA 3' |  |
| BnaC05g34170D-OE | F: 5' CCATAGCCATGCATACTGA 3' |  |
|  | R: 5' TTTCGCCTCTGTGTAATCTG 3' |  |
